# A *DIRIGENT* Gene *GmDIR26* Regulates Pod Dehiscence in Soybean

**DOI:** 10.1155/2024/2439396

**Published:** 2024-04-30

**Authors:** Zheng Wang, Xiaofang Zhang, Rui Hou, Huiying Zhang, Xu Guo, Xiaofei Ma, Aiqin Xu, Hong Zhu, Shuai Li

**Affiliations:** ^1^College of Life Sciences, Qingdao Agricultural University, Qingdao 266109, Shandong Province, China; ^2^Agricultural Service Center in Huji Town, Jinxiang, Shandong Province 272211, China; ^3^College of Agronomy, Qingdao Agricultural University, Qingdao 266109, China; ^4^High-Efficiency Agricultural Technology Industry Research Institute of Saline and Alkaline Land of Dongying, Qingdao Agricultural University, Qingdao 266109, China

## Abstract

Pod dehiscence brings much loss for modern agricultural production, and multiple pod dehiscence components have been identified in many plant species. However, the pod dehiscence regulation factors in soybean are limited. In this study, we investigate the function of *GmDIR26*, a close homologues gene of pod dehiscence genes *GmPdh1*, *PvPdh1*, and *CaPdh1*, in the regulation of pod dehiscence in soybean. The secondary and tertiary structure analysis reveals that GmDIR26 protein has a similar structure with GmPdh1, PvPdh1, and CaPdh1 proteins. Synteny analysis of soybean and chickpea genomes shows that the genomic region surrounding *GmDIR26* and *CaPdh1* might be evolved from the same ancestor, and these two genes might have similar function. *GmDIR26* shows an increased expression pattern during pod development and reaches a peak at beginning seed stage. Meanwhile, *GmDIR26* exhibits high expression levels in dorsal suture and pod wall, but low expression pattern in ventral suture. In addition, *GmDIR26* shows higher expression levels in pod dehiscence genotype than that in pod indehiscence accessions. Overexpression of *GmDIR26* in soybean increases pod dehiscence in transgenic plants, of which the lignin layer in inner sclerenchyma pods is thicker and looser. The expression levels of several pod dehiscence genes are altered. Our study provides important information for further modification of pod dehiscence resistance soybean and characterization of soybean pod dehiscence regulation network.

## 1. Introduction

Pod dehiscence is an essential process in wild soybean (*Glycine soja*) for seed dispersal. The pod of wild soybean opens at the dorsal suture or ventral suture section when it matures and then spreads its seeds to the environment. However, pod dehiscence brings much loss and decreases soybean yield in modern agricultural production [1]. The ancient wild soybean accessions are transited to modern cultivated soybean (*Glycine max*) during evolution, and loss of seed dispersal is an important agronomic trait during soybean domestication [2]. To further improve the pod dehiscence resistance in soybean, it is necessary to investigate the pod dehiscence functional genes. With the development of soybean reference genomes, many soybean functional genes have been identified [3, 4]. In the past decades, the molecular factors involved in soybean pod dehiscence have been analyzed, and several functional genes participating in soybean pod dehiscence regulatory network have been identified [5–14].

*SHATTERING1-5* (*GmSHAT1-5*), which shares a close phylogenetic relationship with *Arabidopsis* pod dehiscence gene *NAC SECONDARY WALL THICKENING PROMOTING FACTOR 1* (*NST1*), is identified as *GmNST1B* [12]. *GmSHAT1-5* is the first identified gene involved in soybean pod dehiscence regulation during domestication [5, 12, 15]. *GmSHAT1-5* activates soybean pod secondary wall biosynthesis and promotes the thickening of fibre cap cells of secondary walls in soybean pod. *GmSHAT1-5* expression pattern is associated with the content of sclerenchymatous cells and shows higher expression levels in fibre cap cells in pod indehiscence lines than that in pod dehiscence accessions. A 20 bp deletion in the promoter region approximately 4.0 kb upstream of *GmSHAT1-5* initiation codon, which destroys the integrity of a GARP protein binding site of “AGAT,” results in the high expression of *GmSHAT1-5* in the pod indehiscence accession and is responsible for the domestication of soybean pod indehiscence [5]. Moreover, *GmNST1A*, which shares 92.8% amino acid similarity to its paralog gene *GmSHAT1-5*, is associated with pod dehiscence in soybean [12]. In addition, *GmRNF1a* interacts with a MADS-box gene *GmAGL1*, which is involved in pod dehiscence regulation, to accelerate pod dehiscence in transgenic *Arabidopsis*. Further investigation reveals that *GmRNF1a* is artificially selected during soybean domestication [16, 17]. *L1*, encoding a hydroxymethylglutaryl-coenzyme A lyase-like domain protein, is responsible for black pods. *L1* plants show more dehisced pods than *l1* because dark pigmentation increases photothermal efficiency [18]. In common bean, *PvMYB26* is located closely to the major QTL of pod dehiscence and is the best candidate gene for pod dehiscence because of its specific differential expression pattern between pod dehiscence and indehiscence genotypes [19]. *VrMYB26a* is located in a hard selective sweep in mung bean genome and shows reduced polymorphism in the promoter region of cultivars [20].

*GmPdh1*, a *DIRIGENT* (*DIR*) gene family member, is another important gene involved in soybean shatter resistance domestication. *GmPdh1* is mainly expressed in the lignin-rich inner sclerenchyma of soybean pod walls and exhibits the highest level at the lignin deposition initiation stage. The alteration of “A” to “T” in the coding sequence of *GmPdh1* results in the change of a lysine amino acid codon to a stop codon, which is responsible for the transition from pod dehiscence to indehiscence in soybean. Knockout of *GmPdh1* using CRISPR/Cas9 improves pod dehiscence resistance in soybean [6, 21]. *GmDIR27*, a close paralog of *GmPdh1*, exhibits increased expression pattern during pod development before full pod stage. Overexpression of *GmDIR27* increases pod dehiscence in soybean, and the expression of soybean *SEEDSTICK*, *INDEHISCENT*, and *ALCATRAZ* homologous genes is altered in *GmDIR27* transgenic plants [22]. In addition, the orthologs of *GmPdh1* have been identified to be involved in the regulation of pod dehiscence in other legumes. For example, *PvPdh1* has been identified to be involved in lignin biosynthesis and associated with pod dehiscence in common bean (*Phaseolus vulgaris*) [13, 23]. *CaPdh1*, the homologous gene of *GmPdh1* and characterized using a RIL (recombinant inbred line) population, is significantly associated with pod dehiscence in chickpea (*Cicer arietinum*) [24]. *VrPdh1* is considered to be a domesticated gene from shatter to shatter resistance in mung bean (*Vigna radiata*) [25].

Although several soybean pod dehiscence-related genes have been identified in recent years, the genes are still limited for further modification of soybean plants. The molecular mechanism of pod dehiscence regulation network still needs further investigation. In this study, we analyzed the function of *GmDIR26*, which shared close homology to the identified pod dehiscence genes *GmDIR27* and *GmPdh1*, in the regulation of pod dehiscence. Our study provides important information for further characterization of soybean pod dehiscence regulation molecular networks.

## 2. Materials and Methods

### 2.1. Plant Materials and Growth Conditions

Williams 82 (W82) and wild soybean PI 468916 were used in this study [3]. For gene expression analysis of *GmDIR26* in development pods, the different growth stage pods were collected from Williams 82 grown in the field in Qingdao, China, including beginning bloom (R1), full bloom (R2), beginning pod (R3), full pod (R4), beginning seed (R5), and full seed (R6) [26]. To analyze the expression of *GmDIR26* in Williams 82 and PI 468916, R5 pods were sampled from plants grown in the field. To investigate the expression of *GmDIR26* in different sections of soybean pods, the dorsal suture, ventral suture, and pod wall of R3, R4, and R5 stage pods were collected from Williams 82 grown in the field. To analyze the expression of *GmDIR26* in transgenic plants, R5 stage pods of *GmDIR26* transgenic lines and Williams 82 grown in pots in the green house were sampled for analysis. The growth conditions were set as follows: 25°C 16 h light/25°C 8 h dark, and the humidity was maintained at 30%.

### 2.2. Phylogenetic Analysis of DIR Orthologs

To identify the relationship of *GmDIR26* with other *DIR* genes, the amino acid sequence of GmDIR26 protein was used as a blast query against TAIR10 (https://www.arabidopsis.org) and Phytozome 13 (https://phytozome-next.jgi.doe.gov/) to search for its homologous genes. The amino acid sequences of *DIR* orthologs from *Arabidopsis*, common bean (*Phaseolus vulgaris*), chickpea (*Cicer arietinum*), lima bean (*Phaseolus lunatus*), cowpea (*Vigna unguiculata*), rice (*Oryza sativa*), maize (*Zea mays*), and wheat (*Triticum aestivum*) were aligned using MUSCLE software (multiple protein sequence alignment) and used to construct a phylogenetic tree using FastTree with default parameters [27]. The iTOL (https://itol.embl.de/itol.cgi) software was used for optimization of the phylogenetic tree [28]. For the sequence alignment of GmDIR26 protein and its homologous genes, the amino acid sequences of these proteins were aligned using MEGA X and then presented in DNAMAN (version 10) [29].

### 2.3. Synteny Analysis of *GmDIR26* Gene Regions

To identify the synteny regions of soybean and chickpea, the genome sequences of soybean and chickpea were obtained from NCBI database (National Center for Biotechnology Information), and then, the genome information was submitted to MCscanX software to identify the synteny regions of soybean and chickpea genomes with default parameters [30]. The synteny regions of soybean and chickpea were connected using gray lines, and the connection between *GmDIR26* and *CaPdh1* was highlighted using a red line.

### 2.4. Protein Structure Analysis of GmDIR26 Protein

The amino acid sequence of GmDIR26 protein was used to analyze its secondary and tertiary structures. For the secondary structure, GmDIR26 was analyzed using PSIPRED software with default parameters [31], and GmDIR26 protein tertiary structure was predicted using AlphaFold 2 software with default parameters [32, 33].

### 2.5. Plasmid Construction and Soybean Transformation

To construct the *GmDIR26* overexpression plasmid, the coding sequences of *GmDIR26* was amplified from the pod dehiscence genotype PI 468916 using specific primers. The PCR products and pPTN1171 plasmid were disgusted using *Xho* I and *Xba* I, and then, the *GmDIR26* fragment and linearity pPTN1171 were ligated using T_4_ ligase as described [34, 35]. The constructed plasmid was transformed into *Agrobacterium* LBA4404 and then transferred into soybean Williams 82 using *Agrobacterium*-mediated transformation method [22]. The *GmDIR26* transgenic plants were identified using PCR and sequencing of *GmDIR26* PCR fragment. And then, the transgenic *GmDIR26* plants were further confirmed using phosphinothricin-N-acetyltransferase/bar rapid test kit (Artron) according to the manufacturer's instructions. The primers are listed in Supplementary Table S1.

### 2.6. Pod Dehiscence Phenotyping

For pod dehiscence analysis, two *GmDIR26* transgenic soybean lines, Williams 82 and PI 468916, were used and grown in the pots under natural conditions in 2021 in Qingdao, China. The full maturity pods (R8 stage) were sampled as described [22]. The collected soybean pods were transferred into an oven, the temperature of which was set at 37°C, to investigate the dehisced pods. After 60 days, the dehisced pods were analyzed, and the percentage of *GmDIR26* transgenic plants and Williams 82 was calculated. For the cross section analysis, soybean-matured pods were used. Cross sections of pod walls were stained with 10% toluidine blue and observed using a microscope (Olympus CX23, China).

### 2.7. RNA Isolation and Gene Expression Analysis

To analyze the expression of target genes, total RNA of soybean *GmDIR26* transgenic plants and Williams 82 samples were isolated using a RNeasy mini kit (Qiagen) according to the manufacturer's instruction. Then, 1.0 *μ*g total RNA for each soybean sample was used for the synthesis of cDNA with SuperScript II reverse transcriptase (Promega) as described by the manufacturer's instruction. The quantitative real-time PCR (qRT-PCR) was performed using ABI QuantStudio®5 (ABI, USA) machine as described [36]. The amplification program was set as follows: 95°C for 5 s and 60°C for 30 s, 40 cycles. The expression level of the analyzed soybean genes was normalized to a soybean *GmCons4* gene [26]. Each soybean sample was analyzed using three biological replicates. The primers used for each gene are listed in Supplementary Table S1.

## 3. Results

### 3.1. Evolutionary Relationship Analysis of *GmDIR26*

**Figure 1:**
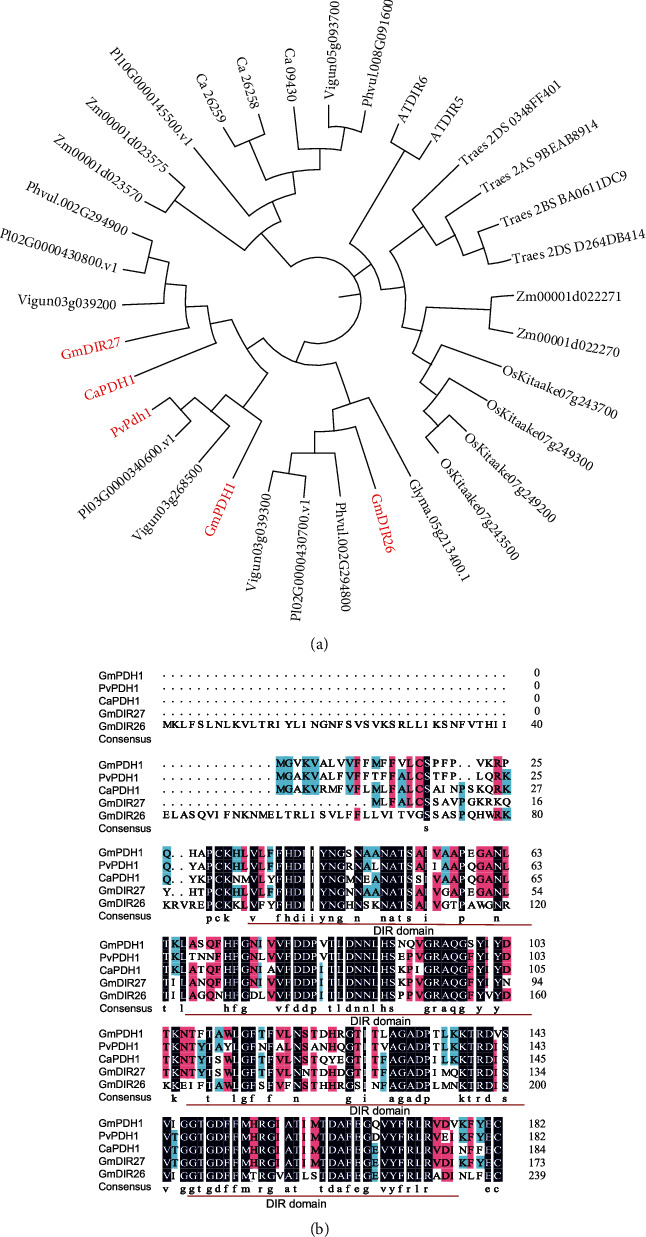
Phylogenetic relationship analysis and sequence alignment of *GmDIR26* and its homologous genes: (a) phylogenetic tree of GmDIR26 and DIR proteins from several plant species (the phylogenetic tree is constructed using amino acid sequences of these proteins); (b) amino acid sequence alignment of *GmDIR26* and pod dehiscence regulation genes *GmDIR27*, *GmPdh1*, *CaPdh1*, and *PvPdh1*. The red line indicates the conserved DIR domain in these proteins.

**Figure 2:**
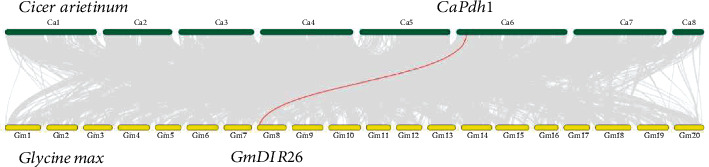
Syntenic relationship analysis of chickpea (*Cicer arietinum*) and soybean (*Glycine max*) genomes. The gray lines indicate synteny blocks within chickpea and soybean genomes, and the red line indicates syntenic regions of *GmDIR26* and *CaPdh1*.

To analyze the evolutionary relationship between GmDIR26 and DIR proteins from other plant species, a phylogenetic tree was constructed using DIR proteins from the model plant *Arabidopsis*; legume crops including soybean, common bean, chickpea, lima bean, and cowpea; and monocotyledons, including rice, corn, and wheat (Figure 1). *GmDIR26* was classified into the same subgroup with *GmPdh1*, *PvPdh1*, *CaPdh1*, and *GmDIR27*, which were involved in pod dehiscence regulation [6, 22, 23], indicating that *GmDIR26* might participate in the regulation of pod dehiscence (Figure 1 and Supplementary Figure S1). To identify the similarity of GmDIR26 and its homologous genes, the sequences of GmDIR26 protein and GmPdh1, PvPdh1, CaPdh1, and GmDIR27 were aligned (Figure 1). We found that all these proteins contained the conserved DIR domain. GmDIR26 protein sequence showed 62.3%, 66.7%, 61.6%, and 58.5% similarities to GmPdh1, GmDIR26, CaPdh1, and PvPdh1, respectively (Figure 1). In addition, the whole genome sequence of soybean was compared with other legumes, and the results revealed that the genomic region surrounding *GmDIR26* showed synteny with that surrounding *CaPdh1*, indicating that *GmDIR26* and *CaPdh1* might be evolved from the same ancestor and have similar functions (Figure 2).

### 3.2. Protein Structure Analysis of *GmDIR26* and Its Homologous Genes

**Figure 3:**
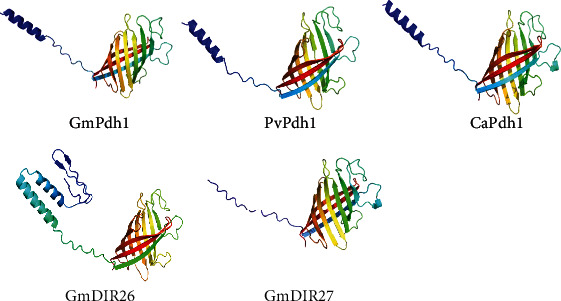
Protein structures of GmDIR26 and its homologous genes.

In legumes, *GmPdh1*, *PvPdh1*, *CaPdh1*, and *GmDIR27* displayed important roles in the regulation of pod dehiscence, and *GmDIR26* showed close relationships with these genes (Figure 1) [6, 22, 23]. To identify the similarity of GmDIR26 with these proteins, we analyzed their secondary and tertiary structures (Figure 3 and Supplementary Figure S1). These proteins showed some similarities in tertiary structures and contained 0-2 alpha helices, 9-11 beta turns, and 9-10 random coils in the conserved DIR domain, respectively (Supplementary Figure S1). However, these proteins also exhibited some differences in tertiary structures. GmPdh1 contained three alpha helices and 11 beta turns, GmDIR27 had one alpha helix and 11 beta turns, PvPdh1 had three alpha helices and 10 beta turns, CaPdh1 contained two alpha helices and 10 beta turns, and GmDIR26 contained four alpha helices and 13 beta turns, respectively (Figure 3).

### 3.3. Expression of *GmDIR26* during Pod Development

**Figure 4:**
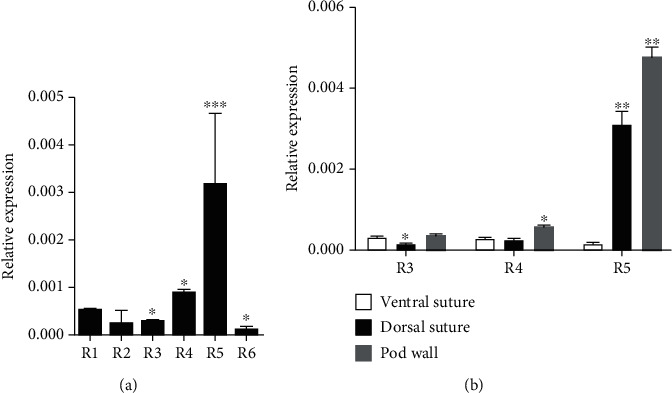
The expression of *GmDIR26* in (a) different development stages and (b) different sections of pods. R1: beginning bloom; R2: full bloom; R3: beginning pod; R4: full pod; R5: beginning seed; R6: full seed. Significant differences relative to the R1 stage are indicated by asterisks. For the expression of *GmDIR26* in ventral suture, dorsal suture, and pod wall, R5 stage pods are used. Significant differences relative to the ventral suture in each stage are indicated by asterisks. The expression of *GmDIR26* is normalized to a *GmCons4* gene. ^∗∗∗^*P* < 0.001; ^∗∗^*P* < 0.01; ^∗^*P* < 0.05.

**Figure 5:**
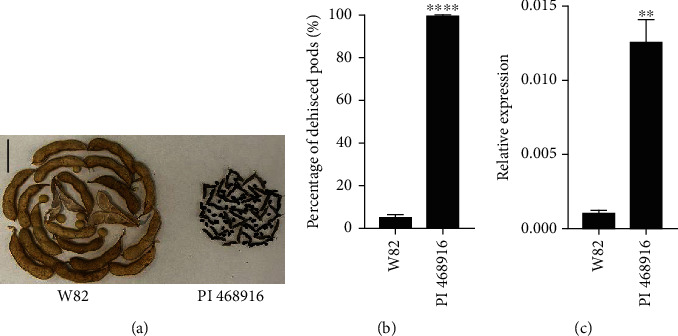
Pod dehiscence and *GmDIR26* expression analysis in Williams 82 and PI 468916: (a) pod dehiscence analysis of Williams 82 and PI 468916; bar = 4 cm; (b) the percentage of dehisced pods in Williams 82 and PI 468916; (c) the expression of *GmDIR26* in Williams 82 and PI 468916. ^∗∗∗∗^*P* < 0.0001; ^∗∗^*P* < 0.01.

The expression of *GmDIR27* and *GmPdh1*, the close homologous genes of *GmDIR26*, displayed different levels during pod development [6, 22], and we analyzed the expression patterns of *GmDIR26* during different pod development stages, including R1 to R6 (Figure 4). The expression of *GmDIR26* showed low levels from R1 to R3 stages and increased from R3 to R4 stages. *GmDIR26* expression reached a peak at R5 stage and decreased at R6 stage (Figure 4(a)), indicating that *GmDIR26* influenced pod development during seed beginning stage. Pod dehiscence indicates that soybean pods open at dorsal or ventral suture. To investigate the potential function of *GmDIR26* in the pod, we analyzed the expression of *GmDIR26* in different sections of pods, including the dorsal suture, ventral suture, and pod wall, which were sampled from R3, R4, and R5 stage pods. *GmDIR26* showed low expression levels in dorsal suture, ventral suture, and pod wall at R3 and R4 stages, respectively (Figure 4(b)). However, *GmDIR26* exhibited high expression levels in dorsal suture and pod wall at R5 stage, but low expression pattern in ventral suture at R5 stage, indicating that *GmDIR26* might be involved in pod development in dorsal suture and pod wall at R5 stage (Figure 4(b)). To compare the expression pattern of *GmDIR26* in pod dehiscence and indehiscence genotypes, we selected PI 468916 and Williams 82 for analysis, which showed 98.7% and 5.6% dehisced pods, respectively (Figures 5(a) and 5(b)). *GmDIR26* showed higher expression levels in PI 468916, indicating that *GmDIR26* might be involved in pod dehiscence regulation (Figure 5(c)).

### 3.4. Overexpression of *GmDIR26* Increased Pod Dehiscence in Soybean

**Figure 6:**
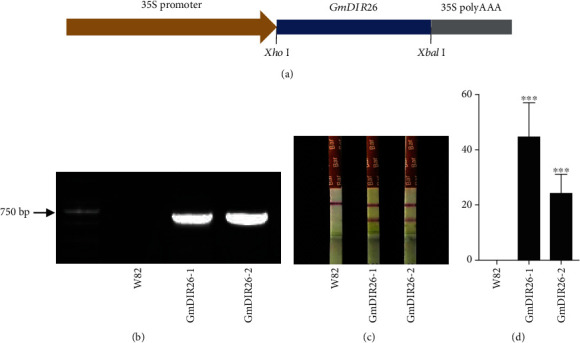
Identification of *GmDIR26* transgenic soybean plants: (a) the schematic diagram of *35S::GmDIR26* in the plasmid; (b) PCR of *GmDIR26* in Williams 82 and transgenic lines using specific primers; (c) bar gene antibody analysis; (d) gene expression analysis of *GmDIR26* in Williams 82 and *GmDIR26* transgenic lines. R5 stage pods are used for analysis. The expression of *GmDIR26* is normalized to the soybean *GmCons4* gene. Significant differences relative to the control Williams 82 are indicated by asterisks, ^∗∗∗^*P* < 0.001.

**Figure 7:**
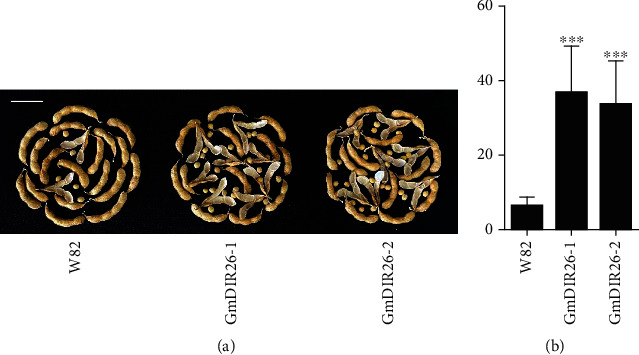
Pod dehiscence analysis of *GmDIR26* transgenic and Williams 82 plants: (a) pod dehiscence phenotype of *GmDIR26* transgenic and Williams 82 plants (full maturity pods are used for analysis; bar = 4 cm); (b) the percentage of pod dehiscence in *GmDIR26* transgenic and Williams 82 plants. Significant differences relative to the control plant Williams 82 are indicated by asterisks, ^∗∗∗^*P* < 0.001.

**Figure 8:**
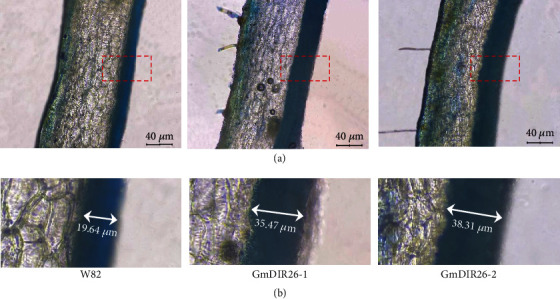
Cross section of pod wall of Williams 82 and *GmDIR26* transgenic soybean plants: (a) the red boxes indicate inner sclerenchyma in pod wall; (b) the arrows indicate the length of inner sclerenchyma in red boxes.

To identify the function of *GmDIR26* in the regulation of pod dehiscence, we constructed a *GmDIR26* overexpression plasmid under the control of the cauliflower mosaic virus 35S promoter and transformed it into the pod indehiscence variety Williams 82 (Figure 6(a)). The transgenic plants were firstly identified with PCR (Figure 6(b)) and then confirmed with bar gene antibody using phosphinothricin-N-acetyltransferase/bar rapid test kit (Figure 6(c)). Then, the expression levels of *GmDIR26* were analyzed in Williams 82 and transgenic plants. *GmDIR26* showed higher expression levels in two transgenic plants than that in the control plant Williams 82 (Figure 6(d)). To investigate the pod dehiscence of Williams 82 and transgenic plants, the matured pods were sampled from soybean plants and transferred into 37°C oven to be analyzed [22]. After 60 days, the two lines of *GmDIR26* transgenic plants exhibited 37% and 33% dehisced pods, respectively, while the pod dehiscence in the control plants was 6.25%, indicating that *GmDIR26* accelerates pod dehiscence in soybean (Figure 7). The anatomical characteristics of *GmDIR26* transgenic pods were analyzed, and the lignin layer in inner sclerenchyma of *GmDIR26* transgenic pods was thicker and looser, while Williams 82 is thinner and compact (Figure 8), indicating that *GmDIR26* has similar function to *GmPdh1* [21].

### 3.5. *GmDIR26* Affects the Expression of Pod Dehiscence-Related Genes

**Figure 9:**
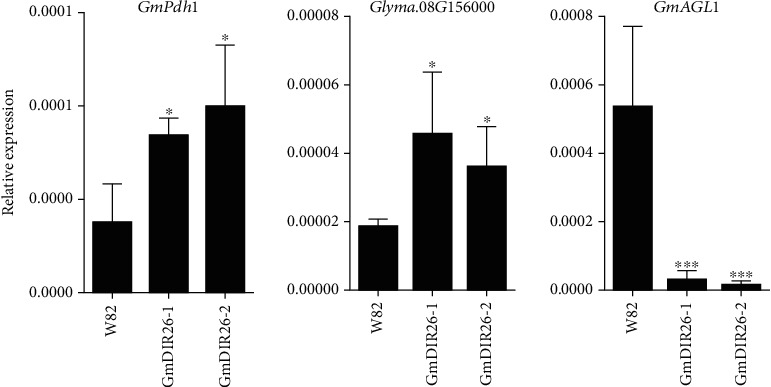
Expression of pod dehiscence-related genes in *GmDIR26* transgenic and Williams 82 plants. R5 stage pods from *GmDIR26* transgenic plants and Williams 82 are used for gene expression analysis. Significant differences relative to the control plants Williams 82 are indicated by asterisks, ^∗∗∗^*P* < 0.001, ^∗^*P* < 0.05.

To investigate the effects of *GmDIR26* on the expression of pod dehiscence genes, we analyzed several functional genes, including *GmPdh1* [6], *GmDIR27* [22], and *GmAGL1*, which accelerated pod dehiscence in plants [16], and *Glyma.08G156000*, the homologous gene of *Arabidopsis* pod dehiscence regulation gene *INDEHISCENT* [37], in the pods of *GmDIR26* transgenic lines and Williams 82 plants (Figure 9). *GmPdh1* and *Glyma.08G156000* slightly increased in *GmDIR26* transgenic plants than that in Williams 82. However, the expression of *GmAGL1* was lower in two *GmDIR26* overexpression lines than that in Williams 82, indicating that *GmAGL1* was suppressed in two *GmDIR26* transgenic plants (Figure 9). *GmDIR27* showed no significant change between *GmDIR26* transgenic plants and Williams 82 (Supplementary Figure S2). These results suggested that *GmDIR26* affected the expression of pod dehiscence-related genes in soybean.

## 4. Discussion

Soybean is an important legume crop and provides essential oil and protein for human food and animal feed. Pod dehiscence brings much loss for the production of soybean, and the investigation of pod dehiscence molecular regulation system will provide genetic resources for soybean modification to improve soybean yield. However, the molecular mechanism regulating pod dehiscence is limited in soybean. In this study, we characterized the function of *GmDIR26* in pod dehiscence regulation and provide important information for further soybean modification.

The homologous genes have the same conserved domains and might have similar functions in plants. *GmSHAT1-5* and its close homologous gene *GmNST1A*, encoding NAC transcription factors, are considered to participate in pod development regulation in soybean [5, 12]. GmPdh1, a DIR domain protein, has been identified to be involved in the regulation of pod dehiscence in the lignin-rich inner sclerenchyma of pod walls [6]. *GmDIR26* and *GmDIR27*, the homologous gene of *GmPdh1*, are important factors to regulate soybean pod dehiscence (Figure 1). Synteny analysis reveals that *GmDIR26* and *CaPdh1* might be evolved from the same ancestor (Figure 2), and they have similar function in the regulation of pod development in legumes. Moreover, DIR proteins are found to regulate the formation of lignan and lignin in plants, which are important components of soybean pod structure, indicating that the DIR proteins are important components in pod development [38–41]. These results suggest that there might be some other *DIR* genes involved in pod dehiscence regulation in legumes, such as *GmDIR19*, which is the close homologous gene of *GmDIR26* (Figure 1).

In soybean, *GmSHAT1-5* and *GmPdh1* have different haplotypes in different varieties, which have distinct functions, and different genotypes of soybean show different degrees of pod dehiscence [5, 6]. Mutation of *GmPdh1* results in the change of a lysine amino acid codon to a stop codon, which leads the change of pod dehiscence to indehiscence [6]. *GmSHAT1-5* and *Gmshat1-5* have some differences in promoter regions; as a result, *GmSHAT1-5* exhibits high expression levels in pod indehiscence genotypes, and *Gmshat1-5* shows low expression pattern in wild soybean, which exhibits pod dehiscence phenotype [5]. *GmDIR26* shows high expression levels in pod dehiscence wild soybean, and overexpression of *GmDIR26* increases the percentage of pod dehiscence in soybean (Figure 7), indicating that the expression level of *GmDIR26* is critical for soybean pod dehiscence, and different expression levels of *GmDIR26* might have different degrees of pod dehiscence. A low expression of *GmDIR26* genotype will be useful for soybean pod indehiscence breeding. Whether *GmDIR26* has different haplotypes, which might affect the expression level of *GmDIR26*, still needs further investigation.

The functional genes might be expressed when its function is needed, and the expression levels of genes in different development stages have a direct relationship with their functions. For example, the expression of soybean growth habit regulation gene *Dt2* is mainly expressed at V2 stage (when the 1st trifoliate leaflet is fully expanded and before the 2ed trifoliate leaflet is unrolled), when it suppresses its downstream gene *Dt1* [42]. The pod dehiscence-related genes are expressed at different stages, and they might participate in different pod development stages in soybean. For example, *GmSHAT1-5* shows low expression level at early pod development stage and reached a high level at approximately 18-day-old pods (approximately R5 stage) [5]. The expression level of *Pdh1* increases at early pod development stage and reaches a peak at 21-day-old pods (approximately R5 stage) [6]. *GmDIR27* shows increased expression pattern during R1 to R4 and reaches a peak at R4 stage [22]. In addition, *GmDIR26* exhibits low expression pattern during early pod development stages and reaches a high expression level at R5 stage (Figure 4). These results indicate that these genes might be involved in pod development at middle growth stages.

In plants, the pod dehiscence regulation system contains complex components to form an effective network, and many related genes are still unknown. The genes regulate the same agronomy trait that might have cooperative or antagonist effect on the phenotype [43]. In soybean, the expressions of *GmPdh1* and *Glyma.08G156000* are slightly increased in *GmDIR26* transgenic plants, indicating that *GmDIR26* might have cooperative effect with these two genes in pod development regulation (Figure 9). However, *GmAGL1* is decreased significantly in *GmDIR26* overexpression lines, suggesting that they might be antagonist in pod dehiscence process (Figure 9). In addition, DIR proteins regulate the production of pinoresinol in plants, which is necessary of the synthesis of lignans and lignin [44, 45]; thus, how *GmDIR26* affects the expression of other pod dehiscence genes still needs further investigation.

## 5. Conclusions

In summary, we identified a DIR protein from soybean, which is the homologous gene of pod dehiscence genes *GmPdh1* and *GmDIR27*. Synteny analysis reveals that *GmDIR26* and *CaPdh1* might be evolved from the same ancestor. The expression of *GmDIR26* shows different expression levels in different development stages and different pod sections. Overexpression of *GmDIR26* increased pod dehiscence by affecting the expression of several pod dehiscence genes.

## Data Availability

Data supporting this research article are available from the corresponding author or first author on reasonable request.

## Supplementary Materials

Supplementary Materials Supplementary Figure S1: secondary protein structures of GmDIR26 and its homologous genes. The red lines indicate the conserved DIR domains in these proteins. Supplementary Figure S2: expression of *GmDIR27* in *GmDIR26* transgenic plants. R5 stage pods from *GmDIR26* transgenic plants and Williams 82 are used for gene expression analysis. Supplementary Table S1: primers used in this study.
