# Supplementary material for: A DIRIGENT Gene GmDIR26 Regulates Pod Dehiscence in Soybean
Source: Int J Genomics. 2024 Apr 30;2024:2439396. doi: 10.1155/2024/2439396 (PMC11074831; doi:10.1155/2024/2439396)
Supplement: Supplementary Materials — Supplementary Figure S1: secondary protein structures of GmDIR26 and its homologous genes. The red lines indicate the conserved DIR domains in these proteins. Supplementary Figure S2: expression of GmDIR27 in GmDIR26 transgenic plants. R5 stage pods from GmDIR26 transgenic plants and Williams 82 are used for gene expression analysis. Supplementary Table S1: primers used in this study. [file 2439396.f1.doc]

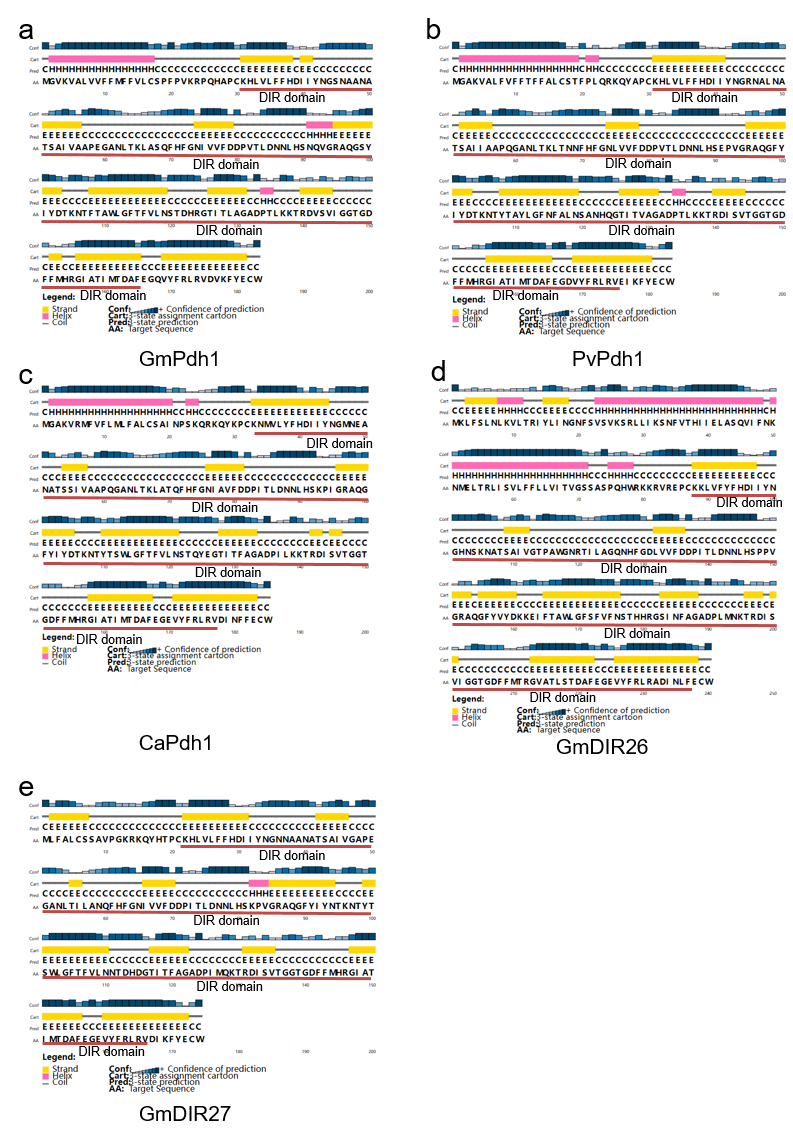


Supplementary Figure S1: Secondary protein structures of GmDIR26 and its homologous genes. The red lines indicate the conserved DIR domains in these proteins.


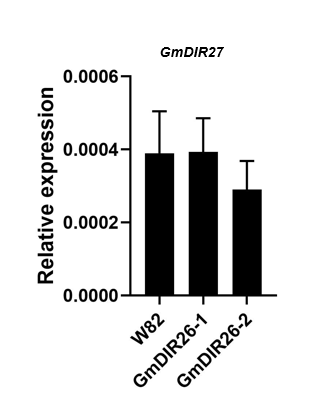


Supplementary Figure S2: Expression of *GmDIR27* in *GmDIR26* transgenic plants. R5 stage pods from *GmDIR26* transgenic plants and Williams 82 are used for gene expression analysis.

| Supplementary Table S1. Primers used in this study | |  |
| --- | --- | --- |
| Gene name | Primer Sequences | Purposes |
| *GmDIR26-F* | CTCGAGATGAAGTTGTTCAGTTTAAA | Plasmid construction |
| *GmDIR26-R* | TCTAGAACTTGTTCGAATGTTGGTGA |
| *GmDIR26-F* | TGGAACTGACGAGGCTCATT | Identification of transgenic plants |
| *GmDIR26-R* | CAACACATGAGCGAAACCCT |
| *GmDIR26-F* | TTGTCTTCAACTCTACCCACCA | qRT-PCR |
| *GmDIR26-R* | GAAGTCACCTGTCCCTCCAA |
| *GmDIR27-F* | GCAAACACTTGGTCCTCTTCTTCC | qRT-PCR |
| *GmDIR27-R* | CACCTTCTGGGGCTCCTACTATTG |
| *GmPdh1-F* | TCGTGGAACCATCACCTTGG | qRT-PCR |
| *GmPdh1-R* | ACATCAACACGAAGCCGGAA |
| *Glyma.08G156000-F* | AATGTGCTACCCCGTTCC | qRT-PCR |
| *Glyma.08G156000-R* | GAGGAGCCATCACAAGCATG |
| *GmAGL1-F* | AGTTCTCATTGCATAGCCATAGA | qRT-PCR |
| *GmAGL1-R* | TGTGTTCTCGATCCGCTTGA |
| *GmCons4-F* | CGGTGGTTCTATCTTGGCATC | qRT-PCR |
| *GmCons4-R* | GTCTTTCGCTTCAATAACCCTA |
